# Mixed pulmonary infection with four isolates of nontuberculous mycobacteria: a case report of *mycobacterium bacteremicum* infection

**DOI:** 10.1186/s41479-022-00100-6

**Published:** 2022-11-05

**Authors:** Morteza Masoumi, Fatemeh Sakhaee, Mohammad Reza Zolfaghari, Samira Tarashi, Fatemeh Rahimi Jamnani, Farzam Vaziri, Seyed Davar Siadat, Abolfazl Fateh

**Affiliations:** 1 Department of Mycobacteriology and Pulmonary Research, Pasteur Institute of Iran, Tehran, Iran; 2 Department of Microbiology, Qom Branch, Islamic Azad University, Qom, Iran; 3 Microbiology Research Center (MRC), Pasteur Institute of Iran, Tehran, Iran

**Keywords:** *Mycobacterium fortuitum*, *Mycobacterium chelonae*, *Mycobacterium mucogenicum*, *Mycobacterium bacteremicum*

## Abstract

**Background:**

A mixed pulmonary infection of *Mycobacterium bacteremicum* and three different isolates of nontuberculous mycobacteria (NTM) is an unusual clinical manifestation and have not yet been indicated. In this case report, we reported four isolates of NTM using phenotypic and genotypic test of pulmonary sample in Tehran, Iran.

**Case presentation:**

We report a case of severe pulmonary disease in a 19-year-old male patient with productive cough, shortness of breath, and low-grade fever for several weeks. The C-reactive protein (CRP) level (80.2 mg/L) and erythrocyte sedimentation rate (ESR) (95 mm/h) were high. The computed tomographic scan indicated bronchiectasis, nodular opacities, consolidation, and cavitary lesions on both sides. The result of purified protein derivative (PPD) test was equal to 15 mm. The sequences of *hsp65, rpoB,* and *16S rDNA* genes indicated more than 99% homology to four isolates of nontuberculous mycobacteria (NTM), including *Mycobacterium fortuitum*, *M. chelonae*, *M. mucogenicum*, and *M. bacteremicum*. We found that all four strains were susceptible to amikacin, cefoxitin, ciprofloxacin, clarithromycin, imipenem, and linezolid. The patient was treated with ciprofloxacin, clarithromycin, and amikacin, along with Montelukast, for five months.

**Conclusion:**

We report a case of severe pulmonary infection by four isolates of NTM. After treatment, the patient reported complete resolution of the signs and a weight gain of 5 kg; also, the CRP and ESR were normal. Nine months after the infection diagnosis, a new CT scan revealed further improvements.

## Background

Nontuberculous mycobacteria (NTMs) have been isolated around the world and are being increasingly identified as human pathogens. Lung disease is the most common manifestation of NTM [1]. Overall, rapidly growing mycobacteria (RGM) are a major cause of NTM infections, particularly in Asia. Among RGM, the most common ones include *Mycobacterium (M.) abscessus*, *M. fortuitum* group and *M. chelonae* [2]. Here, we present a case of severe mixed pulmonary infection, caused by four isolates of NTM.

## Case presentation

The patient was a 19-year-old male with productive cough, shortness of breath, and low-grade fever for several weeks in Iran. His history showed that he kept many birds at home and also fed a cockatiel mouth-to-mouth for several years. He had shown axillary lymph node involvement when he was 16 years old. After seven months, he complained of joint involvement, and two months later, he presented with shortness of breath, sputum, weight loss (8 kg), chest pain, and night perspiration. Bronchoscopy was carried out, while polymerase chain reaction (PCR), acid-fast staining, and culture for *M. tuberculosis* were all negative. Treatment with many drugs, including doxycycline, ceftriaxone, cefixime, co-amoxiclav, was initiated at different intervals; however, it was not successful.

Six months later, the patient’s status gradually deteriorated. He presented with shortness of breath with 92% oxygen saturation in ambient air, besides productive cough; the C-reactive protein (CRP) level (80.2 mg/L) and erythrocyte sedimentation rate (ESR) (95 mm/h) were also determined. The result of purified protein derivative (PPD) test was equal to 15 mm.

**Fig. 1:**
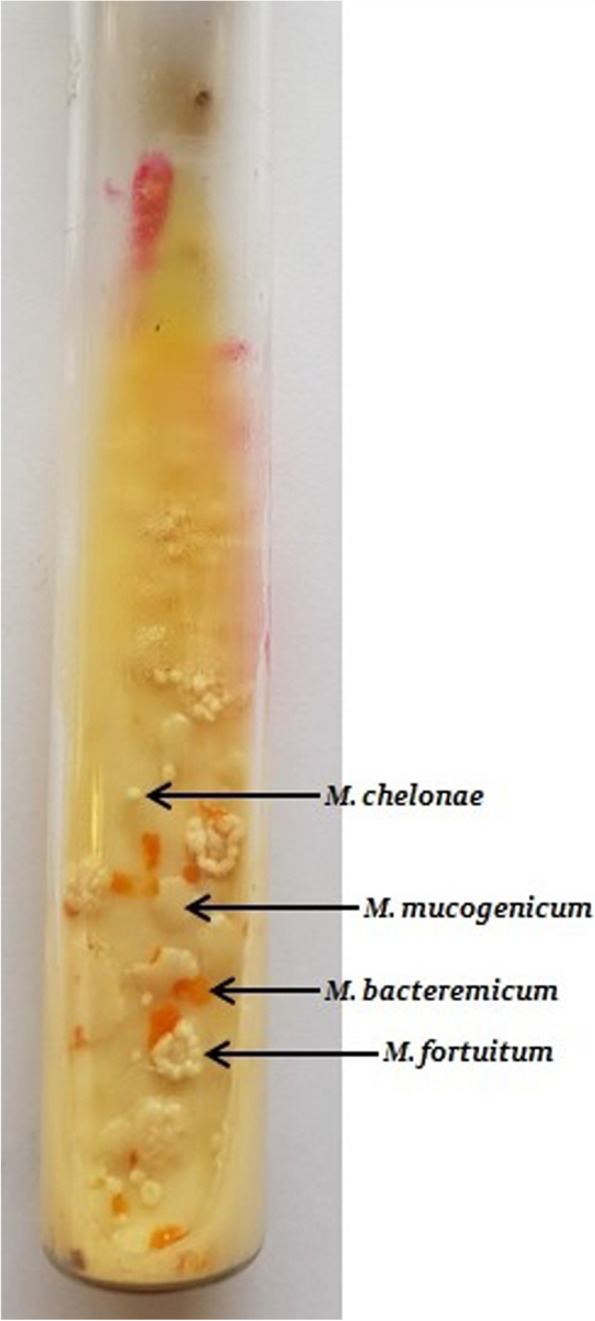
Showing four colonies of nontuberculous mycobacteria over Lowenstein-Jensen media slant

The computed tomographic (CT) scan indicated bronchiectasis, nodular opacities, consolidation, and cavitary lesions on both sides. Newly collected three sputum and three bronchoalveolar lavage samples were sent for further evaluation of the presence of mycobacterial infections. Contrary to previous findings, the results of acid-fast staining and culture on Lowenstein-Jensen medium were positive for all six samples. We observed four different colonies with rapidly growing mycobacteria (one scotochromogen and three nonchromogens) in the Lowenstein-Jensen medium (Fig. 1).

The *hsp65, rpoB* and full *16S rDNA* genes were used for molecular identification, as previously described in the literature [3]. The sequencing results of four isolates of colonies showed more than 99% similarity with *M. fortuitum*, *M. chelonae*, *M. mucogenicum*, and *M. bacteremicum*.

We then performed drug susceptibility testing (DST), according to the Clinical & Laboratory Standards Institute (CLSI) guidelines [4] for amikacin, levofloxacin, clarithromycin, cefoxitin, ciprofloxacin, doxycycline, linezolid, imipenem, minocycline, trimethoprim sulfamethoxazole, and vancomycin. We found that all four strains were susceptible to amikacin, cefoxitin, ciprofloxacin, clarithromycin, imipenem, and linezolid. Based on the susceptibility data for all four NTM isolates, treatment was initiated with ciprofloxacin, clarithromycin, and amikacin, along with Montelukast, for five months. The patient reported complete resolution of the signs and a weight gain of 5 kg; also, the CRP and ESR were normal. Nine months after the infection diagnosis, a new CT scan revealed further improvements. Also, the culture and smear results for the presence of NTM was negative.

## Discussion and conclusion

Generally, mixed NTM infections are correlated with immune suppression, especially in HIV patients. A retrospective study demonstrated mixed infections with other NTM, such as *M. abscessus*, *M. fortuitum*, *M.chelonae*, and *M. simiae* in 29% of *M. avium* complex patients [5, 6]. However, to the best of our knowledge, pulmonary infection, caused by four isolates of NTM isolates, has not been previously reported, particularly with *M. bacteremicum*.

Several studies have suggested that RGM, especially *M. fortuitum*, *M. chelonae,* and *M. mucogenicum*, can cause a variety of diseases, including pulmonary infection [7, 8].

*Mycobacterium bacteremicum* is a scotochromogenic RGM isolated from 10 young patients, two of whom were immunosuppressed. In three and one cases, the strain grew from the blood and the central catheter, respectively [9, 10]; there was no report of pulmonary infection by this isolate. As its name suggests, *M. bacteremicum* is mostly recovered from the blood [9].

In the present case report, our patient had close contact with birds. Infection might have been transmitted through birds, especially when the patient fed the birds orally. However, mycobacteria should be taken seriously by birds living near humans, as they can be a potential reservoir of infections by mycobacteria that are resistant to some antibacterial agents [11]. Because of mixed infection with four types of NTM isolates, DST was performed for the best treatment. The patient responded well to the selected regimen, including ciprofloxacin, clarithromycin, and amikacin. Overall, there is no information concerning the treatment of *M. bacteremicum,* while several reports have shown the successful treatment of *M. fortuitum*, *M. chelonae*, and *M. mucogenicum* with the aforementioned drugs [8, 9]. Considering the complete resolution of symptoms and improvements after appropriate treatment in our patient, this study confirmed the risk of pulmonary infection caused by *M. bacteremicum,* accompanied by three well-known NTM.

Because different NTM may mimic each other, many cases of mixed NTM infection are likely to be missed or misdiagnosed as a single-organism NTM infection, leading to inappropriate antibiotic prescriptions.

## Data Availability

All data generated or analyzed during this study are included in this published article.

## Abbreviations

NTM: Nontuberculous mycobacteria
RGM: Rapidly-growing mycobacteria
PCR: Polymerase chain reaction
CT: Computed tomographic
CRP: C-reactive protein
ESR: Erythrocyte sedimentation rate
WBC: White blood cells
AFB: Acid-fast bacillus
RGM: Rapidly-growing mycobacteria
DST: Drug susceptibility pattern
CLSI: Clinical and Laboratory Standards Institute

## References

[1] Donohue MJ (2021). Epidemiological risk factors and the geographical distribution of eight Mycobacterium species. BMC Infect Dis.

[2] Tang S, Lye D, Jureen R (2015). Rapidly growing mycobacteria in Singapore, 2006–2011. Clin Microbiol Infect.

[3] Davari M, Irandoost M, Sakhaee F (2019). Genetic diversity and prevalence of nontuberculous mycobacteria isolated from clinical samples in Tehran. Iran Microb Drug Resist.

[4] Woods GL, Brown-Elliott BA, Conville PS, et al. Susceptibility Testing of Mycobacteria, Nocardiae, and Other Aerobic Actinomycetes [Internet]. 2nd Ed. Wayne (PA): Clinical and Laboratory Standards Institute; 2011. (CLSI publication / Clinical and Laboratory Standards Institute, No. 31.5.) Available from: https://www.ncbi.nlm.nih.gov/books/NBK544374/31339680

[5] Kim JS, Tanaka N, Newell JD (2005). Nontuberculous mycobacterial infection: CT scan findings, genotype, and treatment responsiveness. Chest.

[6] Kurahara Y, Tachibana K, Tsuyuguchi K, Suzuki K (2013). Mixed pulmonary infection with three types of nontuberculous mycobacteria. Intern Med.

[7] Shrivastava K, Kumar C, Singh A (2020). An overview of pulmonary infections due to rapidly growing mycobacteria in South Asia and impressions from a subtropical region. Int J Mycobacteriol.

[8] Irandoost M, Ghanbari MZ, Sakhaee F (2018). High rates of Mycobacterium fortuitum isolation in respiratory samples from Iranian patients with suspected tuberculosis: is it clinically important?. J Med Microbiol.

[9] Brown-Elliott BA, Wallace RJ, Petti CA (2010). Mycobacterium neoaurum and Mycobacterium bacteremicum sp. nov. as causes of mycobacteremia. J Clin Microbiol.

[10] Tortoli E (2014). Microbiological features and clinical relevance of new species of the genus Mycobacterium. Clin Microbiol Rev.

[11] Ledwoń A, Napiorkowska A, Augustynowicz-Kopeć E, Szeleszczuk P (2018). Drug susceptibility of non-tuberculous strains of Mycobacterium isolated from birds from Poland. Pol J Microbiol.

